# Two entities in pulmonary nodules of a diabetic patient receiving corticosteroid therapy for bullous pemphigoid: an autopsy case report

**DOI:** 10.1186/s12879-022-07566-1

**Published:** 2022-07-07

**Authors:** Reimi Mizushima, Kotaro Haruhara, Nei Fukasawa, Mari Satake, Akira Fukui, Kentaro Koike, Nobuo Tsuboi, Hiroyuki Takahashi, Takashi Yokoo

**Affiliations:** 1 Division of Nephrology and Hypertension, Department of Internal Medicine, The Jikei University School of Medicine, Tokyo, Japan; 2 Department of Respiratory Medicine, Tokyo Medical University Hospital, Tokyo, Japan; 3 Department of Pathology, The Jikei University School of Medicine, Tokyo, Japan

**Keywords:** Autopsy, Corticosteroids, Hemodialysis, Invasive pulmonary aspergillosis, Septic pulmonary embolism

## Abstract

**Background:**

Invasive pulmonary aspergillosis (IPA) is a serious complication occurring in immunocompromised patients, who often show multiple nodular lesions with or without cavitation. Due to high mortality and poor prognosis, the earlier detection and initiation of treatment are needed, while the definitive diagnosis is often difficult to make in clinical settings. Septic pulmonary embolism (SPE) is a complication that occurs in patients with bloodstream infections (e.g., infectious endocarditis). Patients with SPE also present with multiple nodules, nodules with or without cavitation, which are quite similar to the findings of IPA. We herein report an autopsy case that showed multiple nodules due to IPA and infectious endocarditis-related SPE.

**Case:**

A 69-year-old man receiving maintenance hemodialysis due to diabetic nephropathy was admitted with worsening skin rash due to bullous pemphigoid and toxic epidermal necrolysis. He was treated with intravenous methylprednisolone followed by an increased dose of oral prednisolone. On the 6th week of admission, he was diagnosed with infectious endocarditis after the isolation of *Corynebacterium* in blood samples, with a nodule lesion with cavitation in the right lung. Intravenous vancomycin was initiated. After antibacterial treatment, the nodules in the right lung gradually diminished, whereas a nodule with cavitation in the left lung emerged. The nodule in the left lung showed rapid growth along with elevation of serum β-d-glucan and galactomannan antigen. Despite starting treatment with antifungal agents, he died from respiratory failure. An autopsy revealed Groccott staining-positive aspergillus in the left lung, but not in the right lung. We found fibrosis with mitral valve vegetation, indicating a recovery from infectious endocarditis.

**Conclusion:**

Although similar features of nodules with cavitation on CT imaging were shared with SPE and IPA, this case demonstrated that these heterogeneous diseases can occur within the lungs and the distinctly different transitions of CT imaging are helpful for suspecting the presence of multiple pathogeneses.

## Background

Invasive pulmonary aspergillosis (IPA) is an infectious disease with high mortality, that often occurs in immunocompromised patients, including those with hematologic malignancy, allogeneic bone marrow transplantation, solid organ transplantation, human immunodeficiency virus (HIV) infection, and those receiving high-dose corticosteroid therapy [1]. Although the diagnosis of “proven” IPA requires microscopic examination and positive culture from sterile materials [2], it is often difficult to perform bronchoscopy due to the unfavorable status of the patient (e.g., respiratory failure, bleeding tendency, or thrombocytopenia). CT imaging is the fundamental modality to detect IPA. Multiple nodules, nodules with a cavity, and halo sign are characteristic CT findings associated with IPA [3, 4]. Septic pulmonary embolism (SPE) is a complication occurring in patients with bloodstream infections (e.g., bacteremia, catheter-related bloodstream infection, and infectious endocarditis [IE]). Patients with SPE also presented with multiple nodules, nodules with or without a cavity, and halo sign, which are quite similar to the findings of IPA [5, 6]. We herein report the case of a patient undergoing hemodialysis who suffered from IPA and SPE, which both presented lung nodular lesions with a cavity. A postmortem examination revealed worsening of IPA in the left lung and improved SPE in the right lung. This case demonstrated that etiologically distinct diseases, IPA and SPE, may occur within the lungs and that inconsistent transitions of CT imaging are helpful for suspecting the presence of multiple pathogeneses.

## Case presentation

A 69-year-old man was admitted with worsening rashes due to bullous pemphigoid (BP). He had type 2 diabetes, hypertension, and end-stage kidney disease due to diabetic nephropathy. He had been a continuous smoker for approximately 20 years and had quit 40 years previously. He started hemodialysis 4 years before his admission. Six months before his admission, rash emerged on his trunk and upper limbs after the administration of linagliptin, a dipeptidyl peptidase-4 (DPP-4) inhibitor, for his diabetes. Based on a skin biopsy, he was diagnosed with DPP-4 inhibitor-associated BP. DPP-4 inhibitor was discontinued and oral corticosteroid treatment was initiated. Because reducing the corticosteroid dose resulted in a worsening of the rash, he required prednisolone (0.25–0.6 mg/kg/day) thereafter.

After his admission, a second skin biopsy was performed after his skin rash relapsed. The second skin biopsy revealed toxic epidermal necrolysis. Immunoglobulin and intravenous methylprednisolone followed by oral prednisolone (1.0 mg/kg/day) were added. The dose of prednisolone was gradually tapered to 0.4 mg/kg/day during his hospitalization. On the 3rd day of admission, his arteriovenous fistula could not be used due to the worsening of his rash. A temporary blood access catheter was inserted via his right internal jugular vein to perform maintenance hemodialysis. Before and after admission, his glycoalbumin level was in the range of 22–25%, his blood sugar during admission was approximately 150–200 mg/dL, which indicated that his hyperglycemia was relatively well controlled.

**Fig. 1:**
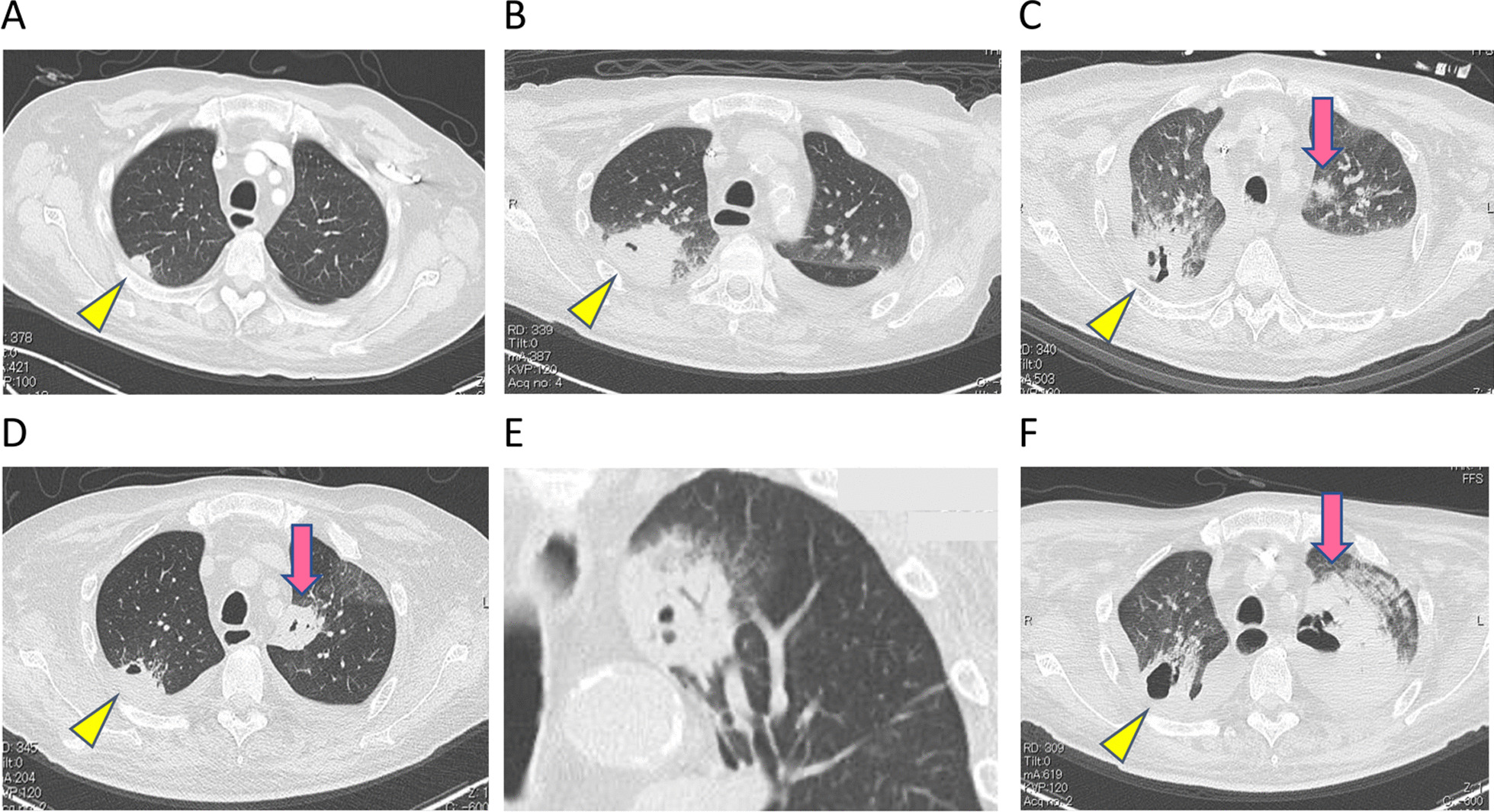
The transition of the patient’s CT imaging. **A**–**D**, **F** Axial CT images on day 28 (**A**), day 41 (**B**), day 53 (**C**), day 61 (**D**), and day 71 (**F**). **A** A nodule in the peripheral region of the right upper lobe (arrowhead). **B** Enlargement of the nodule with newly emerged cavitation in the right upper lobe (arrowhead). **C** A new nodule emerges in the left upper lobe (arrow). **D** The nodule in the left upper lobe is enlarged with cavitation (arrow), whereas the nodule in the right upper lobe is diminished (arrowhead). **E** A coronal CT image on day 61. The nodular lesion in the left upper lobe shows cavitation with the halo sign. **F** Rapid growth of the nodule in the left upper lobe (arrow)

CT showed bilateral deep venous thrombosis of the lower limbs at the time of his admission. Enhanced CT was performed on the 28th day to check them, and a nodule was noted in the peripheral region of the right upper lobe (Fig. 1A). On the 41st day, enlargement of the nodule with newly emerged cavitation (Fig. 1B) was observed. At this time, repeated blood culture tests were consistently positive for *Corynebacterium spp.*. At this time, his plasma was negative for both (1–3)-β-d-glucan and serum galactomannan antigen. Transthoracic echocardiography showed vegetation on the mitral valve with moderate regurgitation. Bilateral cerebral infarction was evident on brain MRI. Based on these findings, he was diagnosed with IE and IE-related systemic embolization involving SPE in the right upper lobe. Intravenous vancomycin was administered.

After 3 weeks of treatment with vancomycin, the nodular lesion in the right upper lobe diminished, whereas a new nodule emerged in the left upper lobe (Fig. 1C). The nodule presented cavitation and a halo sign, which is a macronodule surrounded by a perimeter of ground-glass opacity (Fig. 1D). On the 71st day, CT showed rapid growth of a nodule in the left upper lobe (Fig. 1E). The (1–3)-β-d-glucan level was elevated to > 600 pg/mL and a serum galactomannan antigen test was positive. *Aspergillus sp.* was detected in his sputum culture. Repeated blood cultures were all negative after starting vancomycin treatment. The aspergillus culture of his sputum was totally negative throughout the course. Despite treatment with micafungin followed by amphotericin B, he died from respiratory failure on the 72nd day of admission.

**Fig. 2:**
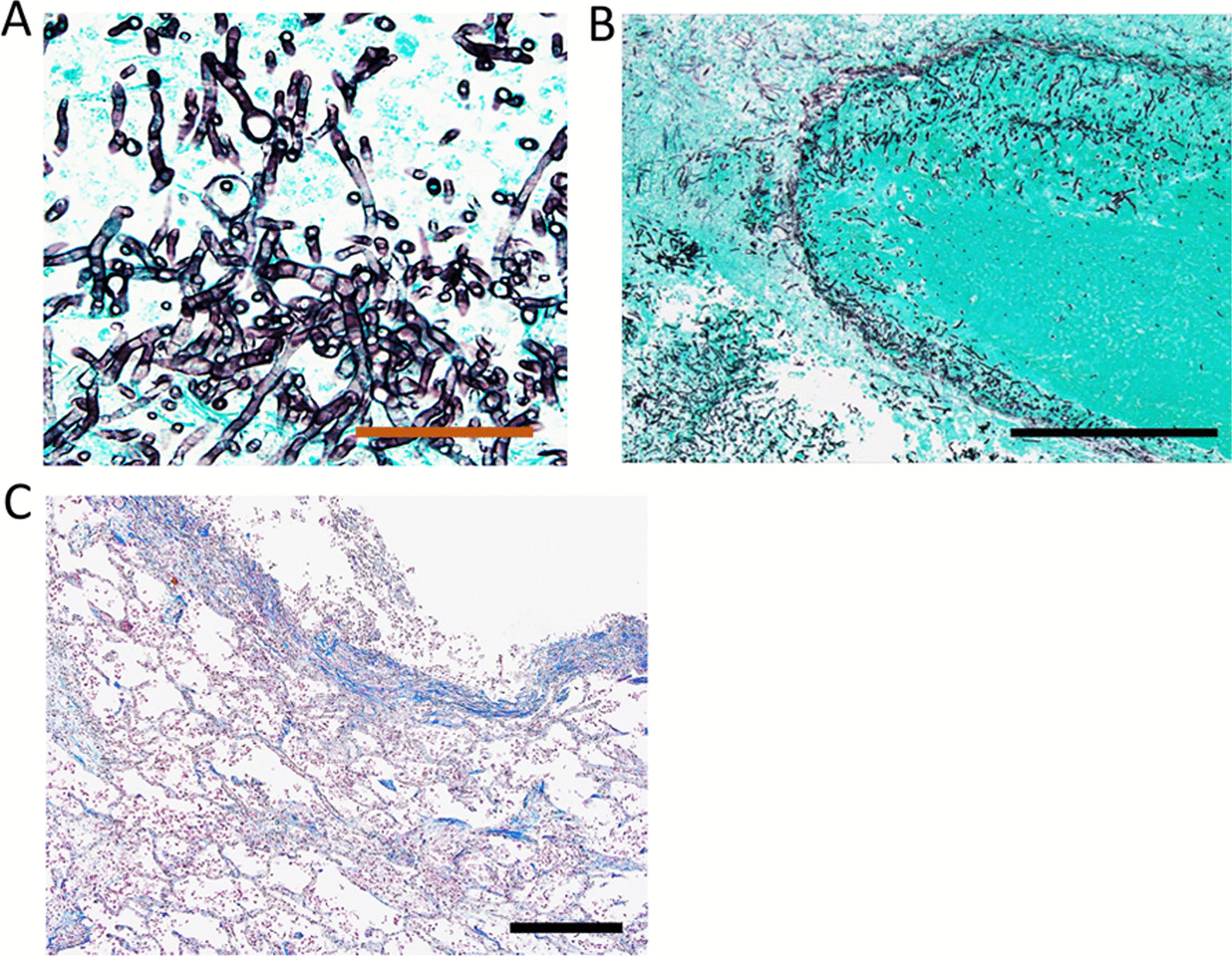
Autopsy findings of the patient. **A** *Aspergillus* hyphae are detected by Grocott staining of a specimen of the left lung nodule. Scale bar = 50 μm. **B** The hyphae invaded vessels in the left lung. Scale bar = 500 μm. **C** The cavity lesion of right upper lobe is surrounded by blue collagen fiber and a small number of inflammatory cells. Scale bar = 500 μm. All images were obtained using an opitical microscope BX53 (Olympus, Tokyo, Japan) and its associated software (cellSens Standard, Olympus)

An autopsy was performed ten hours after his death. In the left lung nodule, *Aspergillus* hyphae was detected by Grocott staining, which had septum and acute angle branches (Fig. 2A). Hyphae were found to be invading into vessels (Fig. 2B) and the same finding was seen in other organs, including the thyroid and right ventricle. These findings are fully consistent with typical histopathological features of IPA. On the other hand, *Aspergillus* hyphae could not be found in the nodule in the right upper lung lobe. A cavity with organized lumen and obstructed vessels was observed. Acute inflammatory cells were partly observed (Fig. 2C). The vegetation on the mitral valve was replaced by fibrosis without inflammatory cells. These findings indicated that the nodule in the right lung lobe was SPE that had partially recovered.

## Discussion and conclusion

We experienced the case of a patient who presented different clinical courses of lung lesions with cavitation due to IE-related SPE in the right lung and IPA in the left lung. Because the left lung nodule caused by IPA emerged 2 weeks after the diagnosis of IE, it was initially suspected to be a SPE caused by IE. After treatment with vancomycin, a blood culture test turned negative and the nodule in the right lung lobe was diminished. On the other hand, the nodule in the left lung lobe showed rapid growth, along with elevation of serum (1–3)-β-d-glucan and galactomannan antigen. Based on the different clinical courses of the nodules in the right and left lung, and the laboratory findings, we considered that the right lung lesion was associated with SPE and lung abscess secondary to IE and that the left lung nodule was caused by IPA. Indeed, autopsy revealed Groccott staining-positive aspergillus in the left lung, but not in the right lung. Although similar lung lesions with cavitation were observed in both the right and left lungs, the different transition of CT images helped us to estimate the etiology of the lung lesions; this was confirmed by autopsy. This case suggests that clinicians should consider the possibility that heterogeneous diseases may occur within the lungs, an assumption based on Hickam’s dictum [7].

When we see a nodule with cavitation, various differential diagnoses should be considered, including lung abscess, lung cancer, pulmonary tuberculosis, and IPA. IPA represents a critical condition, in which delayed treatment leads to increase mortality; however, it is difficult to diagnose at an early stage. The halo sign is a characteristic finding associated with IPA. Greene et al. reported that approximately 60% of IPA patients presented with a halo sign, although the majority of patients in that study had hematological immunosuppressive conditions, in contrast to our patient [3]. The halo sign is pathophysiologically characterized as a nodule of angioinvasive aspergillosis with infarction and coagulative necrosis surrounded by alveolar hemorrhage, which are reported to be imaging features of IPA [8]. Furthermore, the presence of the halo sign was reported to be associate with a significantly better response to antifungal treatment and survival [3]. Other than the halo sign, micronodules, multiple nodules (> 10), and pleural effusion are other imaging features of IPA [3, 9, 10]. On the other hand, SPE contains pathogens that are embolized to the pulmonary artery and which cause focal lung abscess. Typical imaging features of SPE include multiple peripheral nodules in both lungs, cavitation, pleural effusion, and the halo sign [5, 6, 11, 12]. Notably, previous studies—with the exception of a study by Chou et al.—have not comprehensively described the association between SPE and the halo sign [6]. In our case, the IPA in the left upper lobe showed the halo sign, whereas the SPE in the right lobe did not. Although it is not conclusive whether the halo sign is a useful finding for discriminating between IPA and SPE, these two diseases share a number of similar CT features.

IPA is a rare complication in patients with end-stage kidney disease (ESKD). IPA usually occurs in immunocompromised patients, such as those with hematologic malignancy, allogeneic bone marrow transplantation, solid organ transplantation, and late-stage HIV infection [13]. Prolonged and high-dose corticosteroid therapy is an established risk factor for the development of IPA [14]. Some previous cases of IPA with ESKD in patients other than kidney transplant recipients have been reported [15–19]. Most of these IPA cases with ESKD had classical risk factors, such as diabetes or hematologic malignancy [15–17]. Of note, a recent systematic review identified that the most frequently reported baseline clinical factor associated with poor outcomes was renal failure [20]. Careful monitoring for IPA is needed when lung nodules are observed in patients with ESKD or kidney disease, especially those who have classical risk factors for IPA. It would also be important to assess whether the dose and duration of immunosuppressive therapy can be reduced.

The present patient’s serum was negative for (1–3)-β-d-glucan and galactomannan antigen at the time of the diagnosis with IE. These tests turned positive at the latter stage. As the diagnosis of IPA was delayed, the patient did not receive an adequate course of antifungal therapy in time before his death. For the diagnosis of patients at risk of IPA, a galactomannan antigen test shows higher sensitivity and specificity when applied to bronchoalveolar lavage samples than serum samples [21, 22]. Unfortunately, bronchoscopy could not be performed in this case due to the unfavorable status. The performance of a galactomannan antigen test using bronchoalveolar lavage samples would be useful for making a proper and earlier diagnosis and facilitate the initiation of antifungal therapy.

In conclusion, we experienced a case with concurrent IPA and IE-associated SPE, which both presented nodular lung lesions with cavitation, in a diabetic patient under hemodialysis with high-dose corticosteroid therapy. This case indicated that different causes may result in lung lesions with similar features. The different transition of the CT images of the lung lesions suggested that heterogeneous etiologies were involved. IPA is an infectious disease with a very poor prognosis and is associated with high mortality when it occurs in an immunocompromised host with or without classical risk factors. Thus, we should always consider the development of IPA and optimize immunosuppressive therapy of patients with risk factors for IPA.

## Data Availability

Data sharing is not applicable to this article as no datasets were generated or analyzed during the current study.

## Abbreviations

BP: Bullous pemphigoid
DPP-4: Dipeptidyl peptidase-4
ESKD: End-stage kidney disease
IE: Infectious endocarditis
IPA: Invasive pulmonary aspergillosis
SPE: Septic pulmonary embolism
